# Stainless Steel-Supported Amorphous Nickel Phosphide/Nickel as an Electrocatalyst for Hydrogen Evolution Reaction

**DOI:** 10.3390/nano12193328

**Published:** 2022-09-24

**Authors:** Gaoyang Liu, Faguo Hou, Xindong Wang, Baizeng Fang

**Affiliations:** 1 Department of Energy Storage Science and Technology, University of Science and Technology Beijing, 30 College Road, Beijing 100083, China; 2 Department of Metallurgical and Ecological Engineering, University of Science and Technology Beijing, 30 College Road, Beijing 100083, China; 3 Department of Chemical and Biological Engineering, University of British Columbia, 2360 East Mall, Vancouver, BC V6T 1Z3, Canada

**Keywords:** amorphous nickel phosphide, chemical plating, electrocatalysis, hydrogen evolution reaction, water electrolysis

## Abstract

Recently, nickel phosphides (Ni-P) in an amorphous state have emerged as potential catalysts with high intrinsic activity and excellent electrochemical stability for hydrogen evolution reactions (HER). However, it still lacks a good strategy to prepare amorphous Ni-P with rich surface defects or structural boundaries, and it is also hard to construct a porous Ni-P layer with favorable electron transport and gas–liquid transport. Herein, an integrated porous electrode consisting of amorphous Ni-P and a Ni interlayer was successfully constructed on a 316L stainless steel felt (denoted as Ni-P/Ni-316L). The results demonstrated that the pH of the plating solution significantly affected the grain size, pore size and distribution, and roughness of the cell-like particle surface of the amorphous Ni-P layer. The Ni-P/Ni-316L prepared at pH = 3 presented the richest surface defects or structural boundaries as well as porous structure. As expected, the as-developed Ni-P/Ni-316L demonstrated the best kinetics, with $\eta_{10}$ of 73 mV and a Tafel slope of ca. 52 mV dec-1 for the HER among all the electrocatalysts prepared at various pH values. Furthermore, the Ni-P/Ni-316L exhibited comparable electrocatalytic HER performance and better durability than the commercial Pt (20 wt%)/C in a real water electrolysis cell, indicating that the non-precious metal-based Ni-P/Ni-316L is promising for large-scale processing and practical use.

## 1. Introduction

Recently, hydrogen-powered transportation via fuel cells has been attracting much attention. It is necessary to develop economical hydrogen production technology to meet the dramatically increased demand for large-scale applications of fuel cells. Generally, hydrogen can be generated by various strategies such as photocatalytic [1,2,3,4] and electrocatalytic water splitting [5,6,7,8], while proton exchange membrane (PEM) water electrolysis has received widespread attention due to the characteristics of high efficiency, high purity, fast start-up, and strong power strain [9,10].

PEM water electrolysis has two half reactions: the oxygen evolution reaction (OER) [11,12,13] and the hydrogen evolution reaction (HER) [14,15,16]. Currently, one of the challenges lies in the high cost of the precious metal Pt–Pd, used for catalyzing the HER process [17,18] in H_2_- or methanol-fed fuel cells [19,20,21]. In order to further reduce or even avoid the use of precious metals, a large number of non-precious metal catalysts including transition metal sulfides, selenides, phosphides, carbides, nitrides, borides as well as non-metallic carbon materials, or composites of the above components have been broadly investigated [22,23,24]. Among them, transition metal phosphides (TMPs) are currently a class of potential non-precious metal catalysts towards HER, and suitable for acidic environments [25]. Many publications have reported that the catalytic activity and durability, as well as the corrosion resistance of TMPs towards HER, can be boosted through adjustable microstructure, heterostructure, and electronic structure [26,27].

Generally, TMPs are prone to form isotropic crystal structures with exposed crystal facets and unique surface atomic compositions, which are closely related to the adsorption and desorption capacities of H_ads_ during the intermediate step of the HER process. Eventually, both the catalytic reactivity and selectivity of TMPs are influenced [28,29,30]. Among various TMPs, nickel phosphide (Ni-P) has been extensively investigated, such as the crystalline Ni_2_P [31]. The results have shown that the exposed (001) facets of crystalline Ni_2_P contributed significantly higher catalytic activity than other crystal facets [32]. It is widely recognized that the “coordination effect” and “group effect” of the Ni-P formed are conducive to the reception and separation of protons and hydrides, where the exposed surface defects, as well as the structural boundaries with rich coordination of the surface P and Ni atoms, will help boost the intrinsic activity of the HER process [33,34].

It is well known that there are rich surface defects or structural boundaries in the amorphous material due to its randomly atomic compositions and the disorder of atomic arrangement. It has been reported that the unsaturated non-metal elements—e.g., P, S, B—in the amorphous material may result in more unsaturated surface defects or structural boundaries and become high electrocatalytically active sites [24,35,36]. Meanwhile, the amorphous Ni-P may have better corrosion resistance in acidic environments than the Ni-P with crystalline state due to the special interface effects and small size effects of amorphous alloys [26,37]. Therefore, it is believed that constructing amorphous Ni-P with rich surface defects or structural boundaries could be a fascinating strategy to boost intrinsic electrocatalytic activity and enhance electrochemical durability.

Furthermore, the fast mass diffusion and rapid electron transfer in the electrode are also of extreme importance in the HER process [38,39]. At present, the main preparation method of the porous electrode is the catalyst-coated membrane (CCM) method [40]. Although the CCM method has many advantages, the crushing ultrasonic process is not conducive to the maintenance of the original microstructures. In addition, the usage of the ionomer and binder, which are dense, and electronic insulation material will lead to a lack of electronic conductivity and poor mass diffusion channels [41,42]. Recently, porous matrix materials, such as carbon paper (CP), carbon cloth (CC), Ti felt, and stainless steel felt, etc., were used to support the active phase as the integrated porous electrode [36,43]. There are many advantages of the integrated porous electrode. On the one hand, the microstructure of the synthesized catalysts can be maintained and the catalyst with different morphologies can contribute to the improved active area as well as fast mass diffusion; on the other hand, it avoids the use of the ionomer, which is conducive to electron transport and gas–liquid transport between active sites.

In this work, the amorphous Ni-P was deposited on a 316L stainless steel felt (316L felt) to construct an integrated porous electrode via the modified chemical plating. It is well known that the 316L felt is inexpensive, with a similar porous structure to and higher electronic conductivity than CP or CC, which helps the better physical dispersion of the active components [44]. Furthermore, the use of modified chemical plating method not only reduces the size of deposited particles, but also forms porous electrodes, which can further reduce the apparent current density during the electrolysis process and improve the utilization of the catalytic materials. More importantly, the amorphous structure with rich surface defects or structural boundaries can further enhance the adsorption/desorption capacity of the H_ads_ and lead to higher catalytic activities towards the HER. Due to the unique characteristics, the integrated porous electrode prepared at pH = 3 revealed the highest electrocatalytic activity. More importantly, when it was further evaluated in an actual PEM water electrolysis cell, the optimized electrode exhibited comparable catalytic activity towards the HER and showed better stability than that of the commercial Pt (20 wt%)/C.

## 2. Materials and Methods

### 2.1. Materials

All the chemicals and materials were received without further purification. Nafion 212 membrane and Nafion solution (5 wt%) were obtained from DuPont (Wilmington, DE, USA). IrO_2_ was purchased from Beijing Nonferrous Metal Research Institute (Beijing, China), while PTFE suspension (6 wt%) and Pt (20 wt%)/C catalyst were purchased from Shanghai Organic Fluorine Material Research Institute (Shanghai, China) and Johnson Matthey (London, UK), respectively. All other chemicals and materials were received from Sinopharm Chemical Reagent Beijing Co., Ltd. (Beijing, China).

### 2.2. Synthesis of Amorphous Ni-P

Firstly, the 316L felt was pretreated by a series of steps, including rinsing with water and alcohol, boiling in a mixed alkaline solution (80 g L^−1^ NaOH, 20 g L^−1^ Na_2_CO_3_, and 30 g L^−1^ Na_3_PO_4_) for 0.5 h, rinsing with hot water, pickling with 1 mol L^−1^ HCl solution for 0.5 h (pickling helps the removal of the oxide film on the surface of 316L), washing with water, activation with erosion liquid (25 vol% HNO_3_, 25 vol% HCl, 50 vol% H_2_O), and washing with hot water.

Secondly, a Ni interlayer was pre-plated on the surface of 316L felt prior to the chemical plating of Ni-P. A nickel plate was selected as the consumption anode, and the 316L felt worked as the cathode. During the pre-plating, a constant current density of 0.16 A cm^−2^ was applied via a DC regulated power supply in a mixed pre-plating solution (220 g L^−1^ NiCl_2_·6H_2_O, 120 g L^−1^ NiSO_4_·6H_2_O) at 25 °C. The pH value of the mixed pre-plating solution was adjusted to various pH values (i.e., 3, 4, and 5) with 1 mol L^−1^ HCl, and the as-obtained samples are denoted as Ni-316L (pH = 3, 4, 5, respectively).

Finally, the chemical plating of amorphous Ni-P was processed by placing the Ni-316L samples into a mixed plating solution (30 g L^−1^ NiSO_4_·6H_2_O, 30 g L^−1^ NaH_2_PO_2_·H_2_O, 20 g L^−1^ CH_3_COONa, 10 g L^−1^ citric acid, 160 g L^−1^H_2_O) at 80 °C for 6 h. After the completion of the chemical plating, the samples were washed with deionized water and then dried in an oven at 80 °C. A plating time of 0.5 h was used to control the amorphous Ni-P loading at 1 mg cm^−2^. The pH of the mixed plating solution was adjusted with 1 mol L^−1^ NH_3_·H_2_O. The prepared samples were cleaned with ammonia to completely remove the impurities, and then centrifuged again and washed with water and alcohol until there was no Cl^−^ detected. The as-prepared samples were then dried in an oven at 80 °C overnight and the as-prepared samples are marked as Ni-P/Ni-316L (pH = 3, 4, 5, respectively). It should be noted the Ni-P loading of the integrated Ni-P/Ni-316L electrodes used for the water electrolysis cell test was at 5 mg cm^−2^.

### 2.3. Physical and Chemical Characterizations

Scanning electron microscopy (SEM, JSM-7100F; JEOL, Tokyo, Japan) was used to examine surface morphology, while transmission electron microscopy (TEM, FEI TecnaiF30; FEI, Hillsboro, OR, USA), atomic force microscope (AFM, Agilent 5500; Agilent Technologies, Santa Clara, CA, USA), and energy-dispersive X-ray spectroscopy (EDS) were employed, respectively, to examine the microstructures and elemental distribution.

The crystal structures of the samples were confirmed by X-ray diffraction (XRD, Rigaku RINT2400; Rigaku, Tokyo, Japan) and Raman (LabRAMHR Evolution; Horiba, Kyoto, Japan) analysis.

Chemical states of the as-synthesized nanomaterials were investigated with X-ray photoelectron spectroscopy (XPS, Kratos AXIS ULTRADLD; Kratos Analytical, Manchester, UK), and the relative curves were calculated with C 1s (284.8 eV).

### 2.4. Electrochemical Characterizations

VMP2 electrochemical workstation (Bio-logic, Seyssinet-Pariset, France) was used to perform all electrochemical tests, in which a standard three-electrode system was constructed. A Pt sheet (Radiometer Analytical, Lyon, France) was used as the counter electrode and a Cl^−^ free Hg/Hg_2_SO_4_ (REF621, Radiometer Analytical) was used as the reference electrode. The integrated Ni-P/Ni-316L electrodes prepared via the modified chemical plating were used as the working electrode. For comparison, the commercial Pt/C 20 wt% was also deposited on a glassy carbon (GC) substrate (geometric area: 0.5 cm^2^) according to the method reported in our previous work [45], and used as the working electrode for the electrochemical measurements. In this work, the RE (Hg/Hg_2_SO_4_) was calibrated in the pure H_2_-saturated 0.5 mol L^−1^ H_2_SO_4_ solution prior to each measurement, and a clean Pt foil was used as the working electrode. Unless otherwise mentioned, all the potentials referred to the reference electrode were converted to potentials with respect to the reversible hydrogen electrode (RHE) via the following equation: *E*(RHE) = *E*(Hg/Hg_2_SO_4_) + 0.0591 × pH + 0.656.

The double layer capacitance (*C_dl_*) of the catalysts prepared at various pH values was determined from the cyclic voltammetry (CV) measurements at various scan rates according to our previous work [45].

The polarization curves were recorded by the linear scanning voltammetry (LSV) during the potential range of 0.1 to −0.6 V, and the scan rate was 5 mV s^−1^. All the obtained results were in situ iR-corrected (85%).

### 2.5. Water Electrolysis Cell Tests

An ink composed of commercial IrO_2_, polytetrafluoroethylene (PTFE) solution, Nafion^®^, and isopropyl alcohol was sprayed on a PTFE sheet to construct an anode catalyst layer. The as-obtained Ni-P/Ni-316L (pH = 3) was used as a cathode catalyst layer and the gas diffusion layer as well. The anode loading of IrO_2_ was 0.75 mg cm^−2^, while the cathode loading of Ni-P was 5 mg cm^−2^.

For the membrane electrode assembly (MEA) preparation, the Nafion^®^ 212 was pretreated in 5 wt% H_2_O_2_ solution, deionized water, 0.5 mol L^−1^ H_2_SO_4_ solution, and deionized water at 80 °C and 1 h for each step. The CCM coated only with the anodic catalyst layer was achieved by transferring the anodic catalyst layer from the PTFE sheet to the pretreated Nafion 212 under 135 °C, 75 kg cm^−2^ for 3 min. The active area of the MEA was 1 cm^2^, which was fabricated by placing the CCM between a Ti felt and an as-obtained Ni-P/Ni-316L. The performance test was performed in a PEM water electrolysis cell at 80 °C under ambient pressure. Deionized water was fed to the anodic side and cathodic side with a flow rate of 3 mL min^−1^. The polarization curves were recorded by increasing the stepped current density from 0 to 1 A cm^−2^. The stability tests of the catalysts were carried out by chronopotentiometry (CP) at a constant current density under ambient pressure at 80 °C.

## 3. Results and Discussion

**Figure 1:**
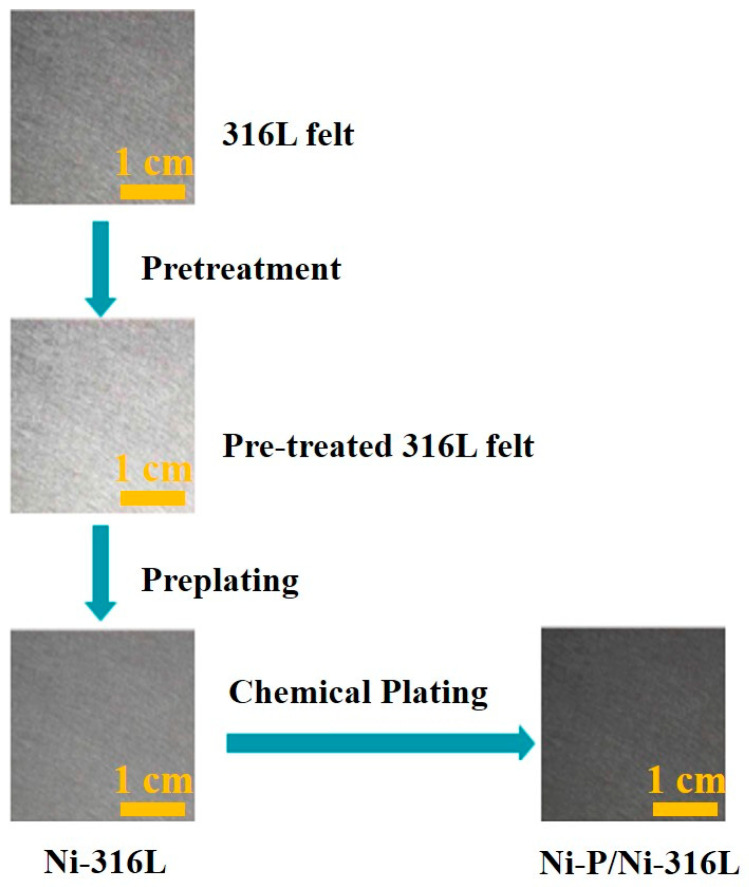
Schematic illustration of synthetic route of the Ni-P/Ni-316L.

The schematic illustration of the synthetic route for the Ni-P/Ni-316L is shown in Figure 1. In this work a Ni interlayer was pre-plated on the surface of 316L felt prior to the chemical plating of Ni-P, in order to completely prevent the oxide film from forming, due to the passivation of the 316L felt, and to provide good adhesion as well. Meanwhile, it was reported that the chemical plating process in an acidic environment is conducive to the formation of amorphous Ni-P, as well as the porous surface morphology [31,46]. In this work, a mixed plating solution was adjusted to various pH values (3, 4, 5) to optimize the morphology of the amorphous Ni-P layer.

**Figure 2:**
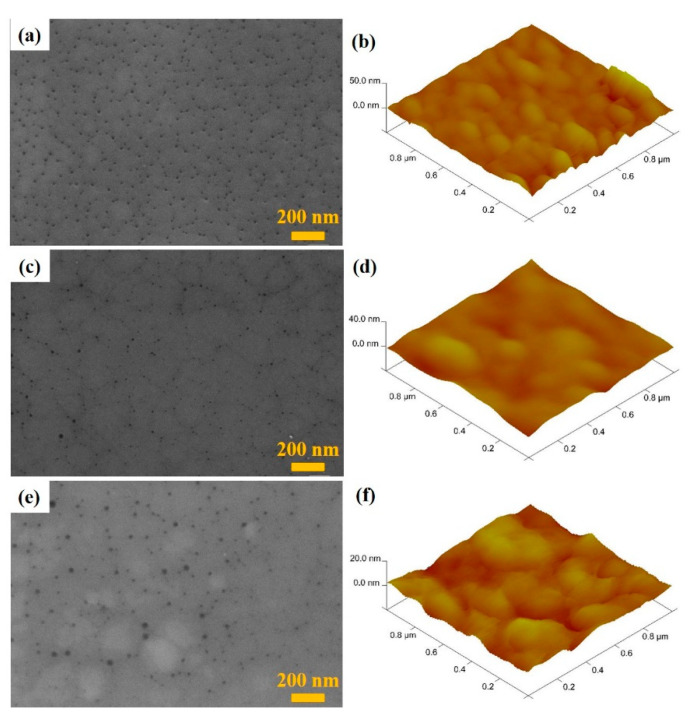
SEM images and AFM images of the Ni-P/Ni-316L samples prepared at different pH values: (**a**,**b**) pH = 3, (**c**,**d**) pH = 4, (**e**,**f**) pH = 5.

Figure 2 shows the SEM images and the AFM images of the Ni-P/Ni-316L samples prepared in the mixed plating solutions with different pH (pH = 3, pH = 4, pH = 5) values. It can be observed that there are many cell-like grains distributed on the surface. Furthermore, a large number of pores emerged along the grain boundaries, which are probably related to the formation of gases during the chemical plating process. By comparing with the SEM images observed for the samples, it can be seen that both the grain size and the porous structure can be affected by the pH of the plating solution. With the pH increased, the pore number was reduced, and the pore size became larger. Meanwhile, as the pH increased, the size of the cell-like grains continued to increase, and the tissue boundary between the grains became narrow, according to the AFM images. It should be noted that the pH increase of the plating solution could accelerate the growth and extension of the cell-like grains, and thus the number of small pores was decreased. Meanwhile, the larger pore size could be ascribed to the violent gas generation under a higher-plating speed. Herein, the AFM images provided the average height and the roughness of the samples. With the pH increase of the plating solution, both the average height and roughness of the deposited layer decreased. It can be seen that when pH was 3, both the finest cell-like grains (around 100 nm) and richest small pores were uniformly distributed, and the roughness was the largest (2.37 nm), indicating that there existed the richest surface defects or structural boundaries. As the pH of the plating solution increased, the cell-like grains agglomerated and the particle size increased to about 200 nm, resulting in a decrease of roughness to 2.01 and 1.54 nm for pH = 4 and pH = 5, respectively. Overall, the grain size, pore size, and distribution, as well as the average height and roughness of the surface, are closely related to the specific surface area of the Ni-P/Ni-316L, which will affect the catalytic activity in the HER process [46].

**Figure 3:**
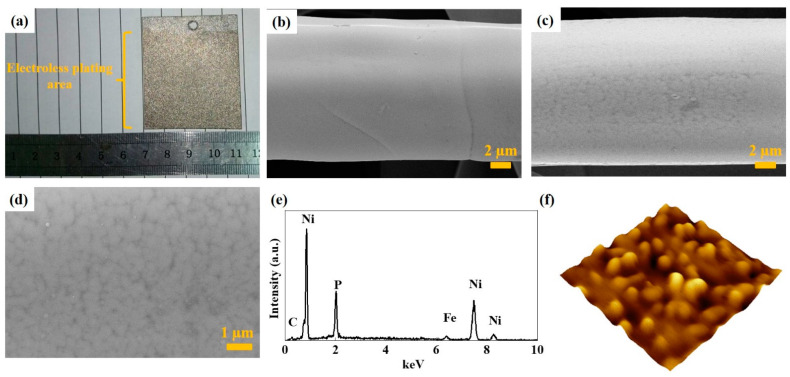
(**a**) Optical photographs of the Ni-P/Ni-316L (pH = 3) used for the water electrolysis cell tests, (**b**) typical SEM images of 316L felt, (**c**) low magnification and (**d**) high magnification of the Ni-P/Ni-316L (pH = 3), (**e**) EDS spectrum of the Ni-P/Ni-316L (pH = 3), and (**f**) AFM image.

Figure 3a is the optical photograph of the integrated Ni-P/Ni-316L (pH = 3) electrode used for the water electrolysis cell tests (the loading of Ni-P increases to 5 mg cm^−2^). It can be seen that the color was changed from the silver of the 316L felt to the silver gray after the chemical plating. Figure 3b shows the SEM image of the original 316L felt, from which it is clear that the surface was quite smooth and no particles were observed. Figure 3c,d presents the low and high magnifications of the SEM images of the integrated Ni-P/Ni-316L electrode, respectively. It can be seen that the Ni-P layer is composed of independent cell-like particles, rather than a dense alloy coating. Even though with a higher Ni-P loading for the integrated Ni-P/Ni-316L electrode compared with the Ni-P/Ni-316L samples in Figure 2, the catalytic layer was still uniform and porous, which is conducive to increasing the specific surface area of the Ni-P, and also facilitates fast mass transports. Meanwhile, the deposition of Ni-P on 316L felt did not lead to a significant change in the fiber diameter, implying the porosity of the integrated Ni-P/Ni-316L was maintained. Figure 3e is the EDS spectra of the integrated Ni-P/Ni-316L electrode, and the presence of characteristic elements Ni and P can be observed. The P content of the prepared sample is basically maintained at a higher level, at about 15 wt%. According to the literature, the Ni-P with high P content is beneficial to the formation of amorphous structures [27]. Figure 3f is a three-dimensional AFM image of the integrated Ni-P/Ni-316L electrode, with a field of view of 20 μm × 20 μm. It further verified that the Ni-P layer consisted of relatively round cell-like particles, which were independent of each other and uniformly distributed, agreeing well with the SEM images.

**Figure 4:**
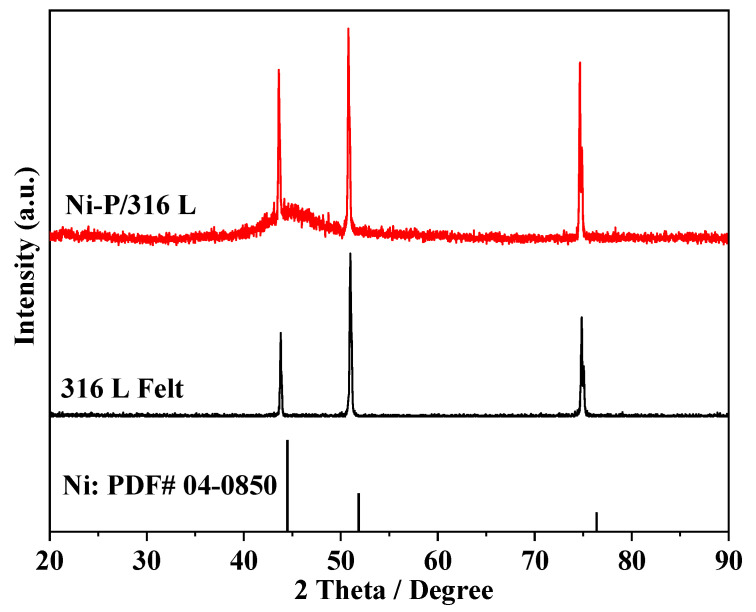
XRD patterns of the Ni-P/Ni-316L (pH = 3) and the 316L felt.

Figure 4 presents the XRD patterns of the Ni-P/Ni-316L (pH = 3) and the 316L felt. Compared with the original 316L felt, only a broad diffraction peak at around 2θ = 44.5°, corresponding to the (111) planes of the fcc Ni (PDF #04–0850), was observed for the Ni-P/Ni-316L (pH = 3). It indicated that the formed Ni-P on the Ni-316L was mainly amorphous, resulting from the presence of P-rich atoms embedded in the Ni lattice, which caused severe lattice distortion [26,37,46].

**Figure 5:**
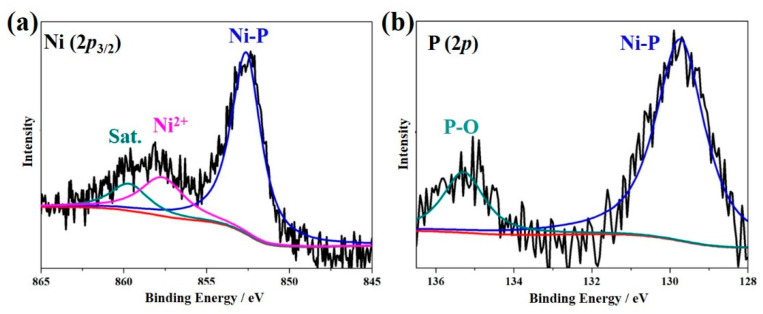
High-resolution XPS spectra of the Ni-P/Ni-316L (pH = 3): (**a**) Ni 2*p*_3/2_, (**b**) P 2*p*.

In order to further probe the components and chemical states of the Ni-P/Ni-316L (pH = 3), Figure 5a,b presents the deconvoluted high-resolution XPS spectra of Ni 2*p* and P 2*p*, respectively. The Ni 2*p*_3/2_ XPS spectrum can be deconvoluted into three peaks. The peak of Ni 2*p*_3/2_ at 852.7 eV was positively shifted by about 0.5 eV compared to the peak of metallic Ni (852.2 eV), which could be attributed to the Ni cations of Ni-P. The Ni 2*p*_3/2_ peak at 857.7 eV corresponded to Ni^2+^ species that have been surface-oxidized to phosphate ions. Meanwhile, the peak at 859.9 eV is a satellite peak of Ni 2*p*_3/2_, which could be corresponded to the divalent species or tri-valent nickel species [47,48]. For P 2*p* in Figure 5b, there exist two peaks at 129.8 eV and 135.3 eV, indicating that two P-containing components were detected. Herein, the peak of P 2*p* at 129.8 eV was negatively shifted by about 0.4 eV compared to the pure P (130.2 eV), and it belonged to the Ni-P bond in in the phosphides. The peak of P 2*p* located at 135.3 eV belonged to P-O bond due to the surface oxidization in nickel phosphate [49]. These evidence confirmed the successful generation of the Ni-P phases on the surface. It has been reported there should be a strong electronic reciprocity between the negatively charged P and the positively charged Ni in Ni-P, which could efficiently promote the electron transfer process, and thus improve the catalytic activity.

**Figure 6:**
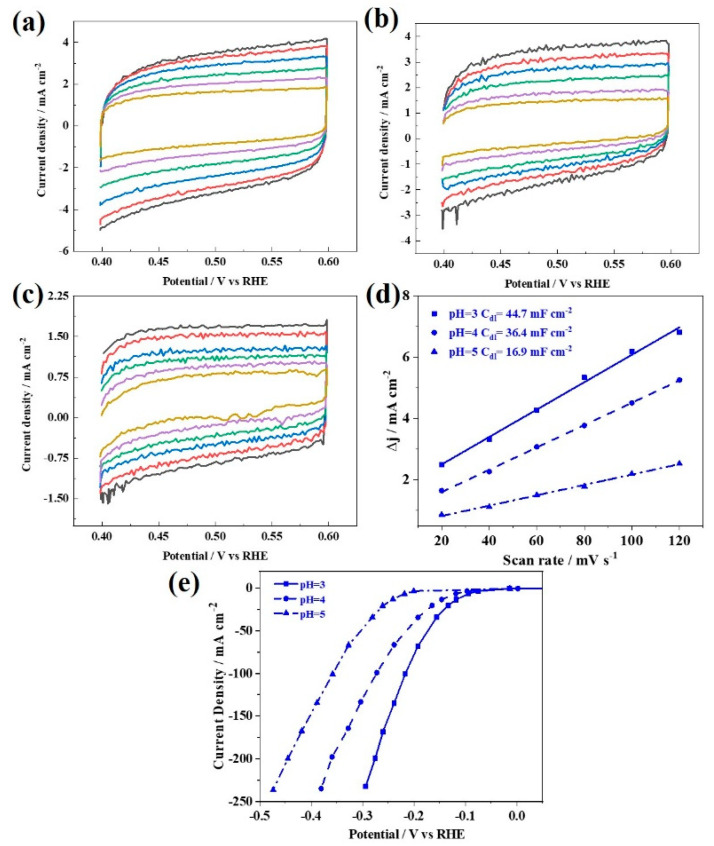
(**a**–**c**) CV curves recorded in the non-Faradaic region at different scan rates. (**d**) Plots of the capacitive currents versus the scan rates: 20 mV s^−1^, 40 mV s^−1^, 60 mV s^−1^, 80 mV s^−1^, 100 mV s^−1^, 120 mV s^−1^. (**e**) *iR*−corrected LSV curves for the Ni-P/Ni-316L samples prepared at different pH values.

As mentioned above, the pH of the plating solution could significantly affect the grain size, pore size and distribution, the average height and roughness of the cell-like grain surface, and finally results in difference in the electrochemical active surface area (ECSA). Herein, the electrochemical double-layer capacitance (*C_dl_*) was used for the qualitative comparison of the ECSA of the Ni-P/Ni-316L samples prepared at various pH values. It was reported that the *C_dl_* should correlate with the number of active sites [50]. Figure 6a–c presents the CV curves recorded in the non-Faradaic region at different scan rates. Figure 6d shows the plots of the capacitive currents versus the scan rates for the Ni-P/Ni-316L sample prepared at different pH values. It should be noted that the *C_dl_* of the Ni-P/Ni-316L obtained at pH = 3 is much higher than those of pH = 4 and pH = 5, implying an obvious increase of the number of the catalytically active sites for the Ni-P/Ni-316L. It could be explained by the better dispersion of the Ni-P particles on the 316L felt due to the higher roughness. Figure 6e presents the steady-state polarization curves of the samples recorded in 0.5 mol L^−1^ H_2_SO_4_ solution at 25 °C and the sweep speed of 1 mV s^−1^. It can be seen that a more significant improvement in the catalytic activity is observed for the Ni-P/Ni-316L obtained at pH = 3. It can be deduced that the finer grain size as well as the porous structure not only ensured that the Ni-P was well-distributed, but also provided sufficient mass transport on the subsurface. In the literature, porous nanostructures facilitate fast mass transport and enhanced cycling stability, which have been frequently reported for diverse catalysis and electrochemical energy-related applications [51,52,53,54,55,56,57].

**Figure 7:**
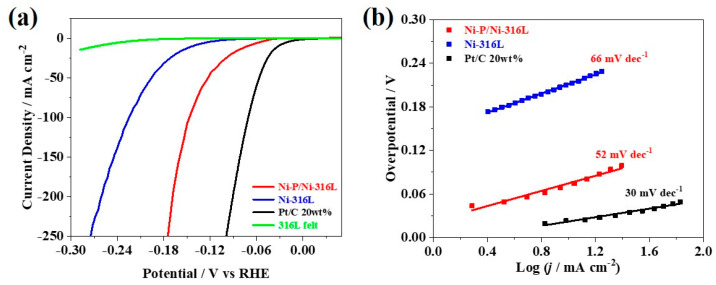
(**a**) *iR−*corrected LSV curves, (**b**) Tafel curves for the different catalytic materials.

Based on the optimized pre-plating Ni interlayer, the integrated Ni-P/Ni-316L electrode with a high Ni-P loading (5 mg cm^−2^) was further prepared for the water electrolysis cell. Figure 7a presents the polarization curves of different electrodes. The overpotential corresponding to a current density of −10 mA cm^−2^ and −100 mA cm^−2^ was defined as $\eta_{10}$ and $\eta_{100}$, respectively. As shown in Figure 7a, the amorphous Ni-P/Ni-316L shows much better HER catalytic activity than that of the Ni-316L and bare 316L felt, and the $\eta_{10}$ and $\eta_{100}$ of the Ni-P/Ni-316L are only 73 mV and 147 mV, respectively. It indicates that the HER activity of the electrode can be significantly promoted via the construction of amorphous Ni-P on the surface of the support material. Although so, there is still a catalytic activity gap between the Ni-P/Ni-316L (pH = 3) and the commercial Pt/C 20 wt%, which has the $\eta_{10}$ and $\eta_{100}$ of 32 mV and 71 mV, respectively. The Tafel slope reflects the reaction mechanism of the HER process, and it is generally used to determine the rate-limiting step of the HER [58,59]. Figure 7b shows the Tafel curves for the different catalysts. Generally, the Ni-P/Ni-316L exhibited more excellent kinetic parameters than that of the Ni-316L and bare 316L felt, and the Ni-P/Ni-316L showed a smaller Tafel slope (ca. 52 mV dec^−1^) than that of the Ni-316L (ca. 66 mV dec^−1^). While the Tafel slopes of the Ni-P/Ni-316L and Ni-316L are located between 39 mV dec^−1^ and 120 mV dec^−1^—indicating that the HER processes on the catalytic surface are in line with the Volmer–Heyrovsky mechanism, where the Volmer process (H^+^ + e^−^ → H_ads_) is the rate-limiting step of the entire HER—this is quite different from the commercial Pt/C 20 wt% in the Volmer—Tafel process, of which a smaller Tafel slope (ca. 30 mV dec^−1^) was obtained [59]. It should be noted that the as-prepared amorphous Ni-P/Ni-316L (pH = 3) in this work could deliver a current density of 250 mA cm^−2^ at a relatively low overpotential in comparison with some HER catalysts reported in the literature, and it is proved that the integrated Ni-P/Ni-316L electrode can be a potential component to replace the Pt-based cathode in a real water electrolyzer. The non-precious metal-based Ni-P/Ni-316L materials are simple in preparation and are also low-cost, making it promising for large-scale processing and practical use.

**Figure 8:**
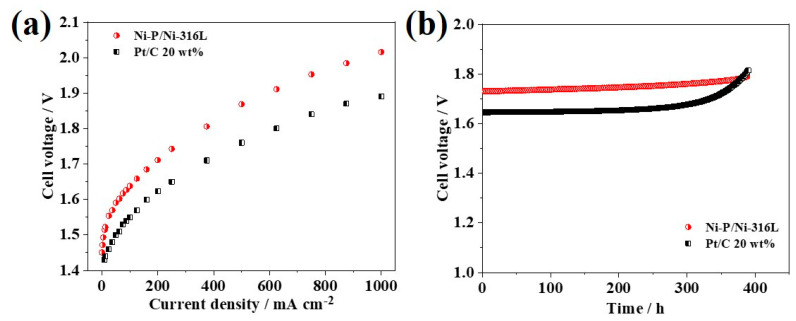
(**a**) I–E curves of the PEM water electrolysis cells with different cathodes, (**b**) stability of PEM water electrolysis cells with the current density of 200 mA cm^−2^.

The Nafion 212 membrane was used as the electrolyte, the MEAs consist of commercial IrO_2_ as the anode, and various cathodes made of the Ni-P/Ni-316L and Pt/C 20 wt% were prepared and evaluated in a homemade water electrolysis cell. In the current study, the anode catalytic layer, the IrO_2_ loading, and fabrication method were kept the same in order to further study the influence of different cathodes. Figure 8 presents the PEM water electrolysis cell performance obtained under ambient pressure at 80 °C, and the cell voltages of the electrolysis cell of MEAs prepared with Ni-P/Ni-316L and Pt/C 20 wt% were 2.02 V and 1.89 V at a current density of 2 A cm^−2^, respectively. It can be seen that there was no transport hindrance up to a current density of 1 A cm^−2^ for all testing cathodes. In this work, the non-noble metal cathode Ni-P/Ni-316L showed a comparable performance to the Pt/C 20 wt%. Even though the cell voltage that demanded to deliver a current density of 1 A cm^−2^ was higher, it still could meet the demand of the application in the water electrolysis cell.

The durability of cathodic hydrogen evolution materials in acidic environments is also an important parameter to meet the demanding targets of practical applications in water electrolyzers [17,34]. Herein, the electrochemical stability of each cathode was evaluated by chrono-potentiometric (CP) method at a constant current density of 200 mA cm^−2^ in the water electrolysis cell at a temperature of 80 °C and under atmospheric pressure. As shown in Figure 8b, the cell with Ni-P/Ni-316L presented reasonably good stability for 400 h. The potential required to achieve the current density of 200 mA cm^−^^2^ increased from 1.73 V to 1.79 V, and the degradation rate of cell voltage was about 0.15 mV h^−1^. In contrast, the cell with Pt/C 20 wt% cathode exhibited worse stability. The potential required to achieve the current density of 200 mA cm^−2^ increased significantly from 1.65 V to 1.81 V, and the decay rate of cell voltage was about 0.40 mV h^−1^. It should be noted that there could be various factors that affect the stability of the water electrolysis cell tests. Importantly, it is believed that the amorphous Ni-P phase, the stable electrode surface microstructure, composition, as well as the good adhesion of the Ni/Ni-P layer could contribute to the superior stability of the integrated Ni-P/Ni-316L electrode. For Pt/C 20 wt%, it has been reported in various literature that the performance loss could be ascribed to the migration and detachment of Pt nanoparticles from the carbon support when a cathodic overpotential is applied. Overall, the Ni-P/Ni-316L exhibited comparable performance to the commercial Pt/C 20 wt% and showed better stability in a real-water electrolysis cell.

## 4. Conclusions

In this work, an integrated porous electrode consisting of amorphous Ni-P with a Ni interlayer was constructed on a 316L stainless steel felt via a modified chemical plating method. The pH of the plating solution was investigated and adjusted to optimize the grain size, the average height, and roughness, as well as the pore size and distribution of the Ni-P layer. SEM, EDS, AFM, XRD, and XPS confirmed that the amorphous Ni-P with rich surface defects or structural boundaries was formed, and the Ni-P catalytic layer was composed of porous, independent cell-like grains, rather than a dense alloy coating. Generally, the Ni-P/Ni-316L (pH = 3) exhibited better kinetic parameters than that of the Ni-316L and bare 316L felt with a smaller Tafel slope. When further evaluated in a homemade water electrolysis cell, the cell voltages at a current density of 2 A cm^−2^ with Ni-P/Ni-316L and Pt/C 20 wt% were 2.02 and 1.89 V, respectively. Meanwhile, the Ni-P/Ni-316L showed better stability for 400 h with a low cell voltage decay rate (about 0.15 mV h^−1^) under the current density of 200 mA cm^−2^.

## Data Availability

Data will be available upon request from the corresponding authors.
